# Transient Neuropathic Pain Following Mechanically Assisted Manipulation of the Spine: A Clinical Case Study

**DOI:** 10.7759/cureus.42912

**Published:** 2023-08-03

**Authors:** Diogo Garcia, Eric Nottmeier, Stephen Pirris

**Affiliations:** 1 Neurosurgery, Mayo Clinic, Jacksonville, USA

**Keywords:** chiropractic therapy, musculoskeletal manipulation, pain, manipulation, spine

## Abstract

We describe the case of a patient developing acute neuropathic pain in the sciatic nerve distribution following spinal manipulation. Manipulative treatment with an Activator Adjusting Instrument (AAI) was recommended and performed. Within 24 hours, the patient developed severe 10/10 pain originating from the left gluteal area at the site of one of the activator deployments with radiation all the way down his left leg to the foot. He was able to maintain distal left leg strength and sensation. Relief was achieved with subsequent physical therapy techniques to relax his deep gluteal muscles, raising the hypothesis of temporary injury to the deep gluteal muscles, with painful contractions resulting in gluteal region pain as well as sciatic nerve inflammation as the nerve passed through that region. This clinical case illustrates some of the perils and risks of spinal manipulation, particularly in the elderly, and the need for careful patient selection.

## Introduction

Chiropractic manipulation of the spine with the use of devices, including the Activator treatment, offers the possibility of controlled speed and thrust with potential benefits in comparison with manual manipulation [1-3]. Complication reports associated with its use are sparse [2,3]. We report the case of a 91-year-old male who presented with low back pain and was submitted to Activator treatment, with the onset of transient neuropathic pain in the sciatic nerve distribution within one day of treatment. Subsequently, the patient developed insomnia, confusion, and adrenal gland dysfunction in response to changes in steroids, gabapentin, and other drugs, thus highlighting some nuances of managing elderly patients with back pain. We found no prior reports of neuropathic pain resulting from Activator treatment.

## Case presentation

**Figure 1:**
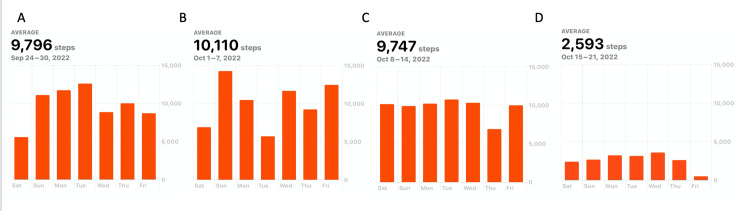
Average number of daily steps. Average daily steps prior to intervention (A) from September 24 to 30, (B) from October 1 to 7, (C) from October 8 to 14, and (D) following intervention from October 15 to 21.

A 91-year-old male with good premorbid status was in his prior state of health until he developed right-sided low back pain after playing golf. He had been playing golf at least two times per week. The pain was located in the right paraspinal region between the rib cage and iliac crest and was rated as 8/10 in severity, with a significant impact on his daily activities. However, the patient continued to be active and was still taking approximately 10,000 steps/day (Figure 1).

**Figure 2:**
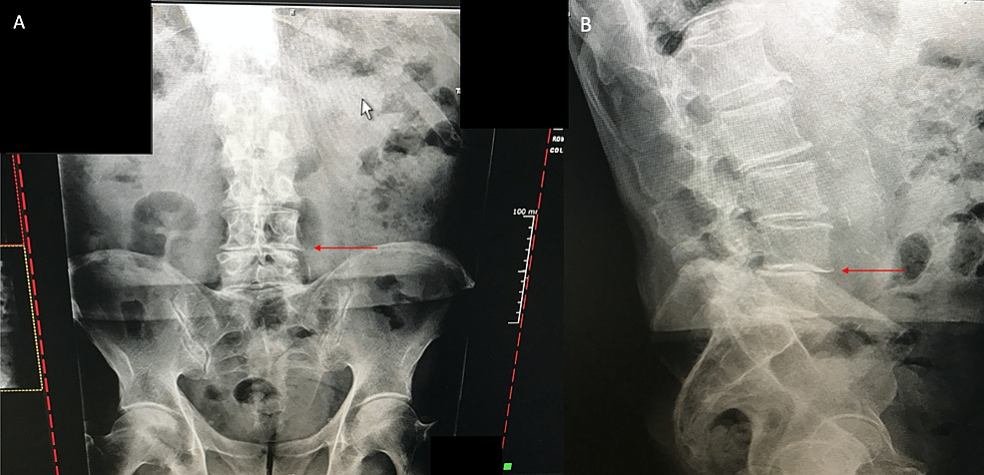
Patient lumbar X-rays. Standing lumbar AP view (A) and lateral view (B). The red arrow indicates L4-5 disc degeneration with grade 1 spondylolisthesis. AP: anterior-posterior.

**Figure 3:**
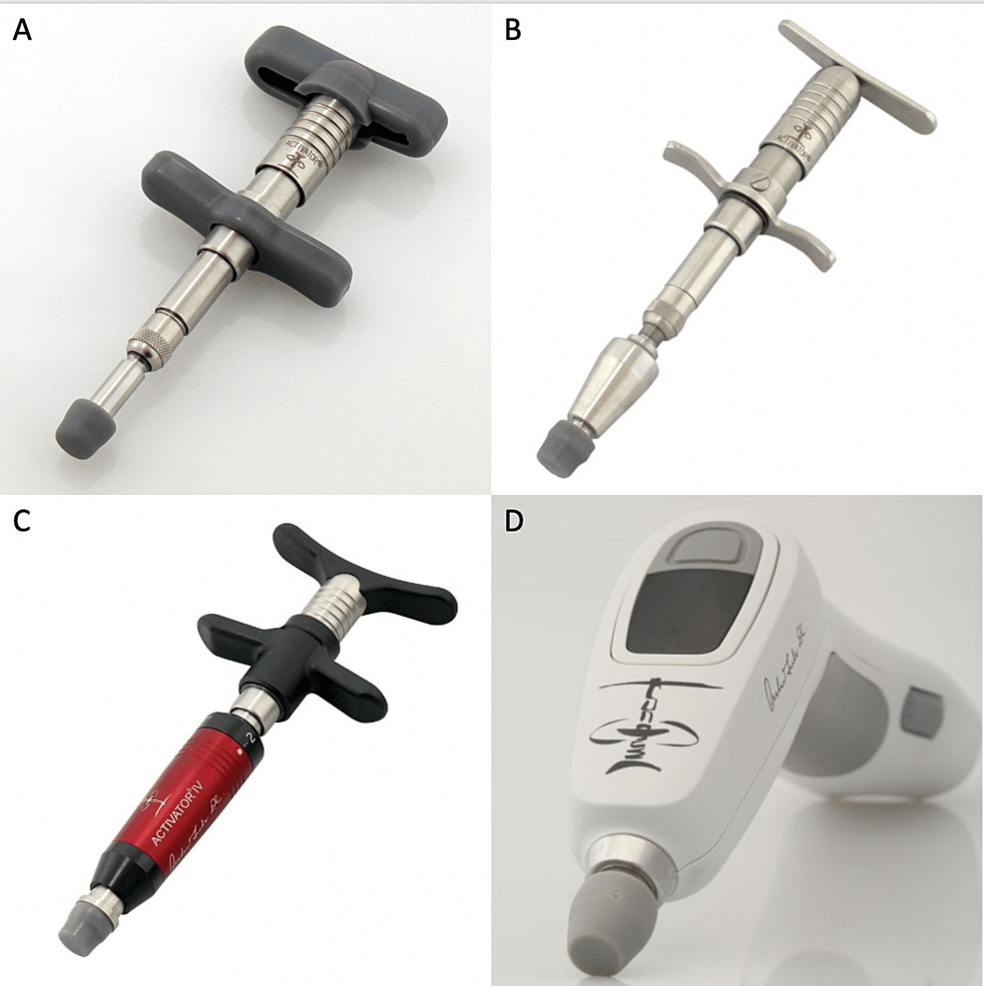
Representative examples of Activator device. (A) Activator I, (B) Activator II EZ Grip, (C) Activator IV EZ Grip, and (D) Activator V.

There was no pain radiating down his leg, and he never had any pain on the left side. Twisting motions exacerbated the right-sided back pain. Rest, including sitting, provided relief. Lumbar flexion and extension movements did not significantly affect the right-sided back pain. Two weeks after the onset of pain, he consulted a local chiropractor. His first two visits included stretches and transcutaneous electrical stimulation treatments, which were effective in relieving his back pain to 0/10. At his second visit, anterior-posterior (AP) and lateral lumbar X-rays were obtained, and pertinent findings included L4-5 disc degeneration with grade 1 spondylolisthesis, mild left convex coronal scoliosis, and slight pelvic obliquity (Figure 2). It was reported that his scoliosis and pelvic obliquity were creating a misalignment that would lead to the recurrence of his pain, so manipulative treatment with an Activator Adjusting Instrument (AAI) was recommended and performed at his third visit (Figure 3). During this treatment, three applications of the AAI were performed bilaterally. The applications were bilateral (1) over the sacroiliac joint, (2) gluteal area, and (3) paraspinal region just above the iliac crest. His back pain remained at 0/10. However, approximately 24 hours after the Activator treatment, he developed severe 10/10 pain originating from the left gluteal area at the site of one of the activator deployments with radiation all the way down his left leg to the foot. He was able to maintain distal left leg strength and sensation.

Due to his inability to sleep, eat, or function due to severe left leg pain even during rest, he presented to the ER and was treated with intramuscular injections of Toradol and Decadron that transiently relieved the pain. He was prescribed gabapentin but did not tolerate the mental status side effects even at a low dose. His pain responded well to further doses of NSAIDs, and his corticosteroid taper was extended to two weeks. Within five days, his pain at rest was limited to the gluteal area, but the radiating pain recurred with any attempt at limited ambulation. In a follow-up discussion with his chiropractor, he was offered further manipulative treatments, but the patient deferred. He was then referred to physical therapy. The physical therapist worked to relax his deep gluteal muscles with massage and electrical stimulation. This provided good relief for his gluteal region and radiating pain almost immediately. The pain recurred, and he did require multiple sessions with the therapist to more completely alleviate his pain. He took two months to regain his stamina and walking endurance. He continued to have difficulty walking due to gluteal region discomfort and a feeling of pelvic instability that was not present prior to the chiropractic manipulation. Endocrinology evaluated the patient and observed adrenal gland dysfunction attributed to the corticosteroids that were required to help the pain. 

## Discussion

While absolute causality in this case could not be demonstrated, temporal relationship and biologic plausibility, including the onset of pain within one day after treatment with AAI in the sciatic nerve distribution without prior history of similar symptomatology, correlate exposure to this complication. Further, pain was noted to localize to the treatment area and radiate in the sciatic nerve distribution. Based on the relief achieved with subsequent physical therapy techniques to relax his deep gluteal muscles, it is plausible that the device caused a temporary injury to the deep gluteal muscles, with painful contractions resulting in gluteal region pain as well as sciatic nerve inflammation as the nerve passed through that region. The question remains whether any manipulation was truly necessary or indicated as part of his initial chiropractic treatment plan. Given that complications associated with similar practices are not often reported in the literature, this case highlights important considerations to be made in the elderly given the potential impact of transient/permanent neuropathic pain in that population subset.

The Activator device, also known as the AAI, is also referred to as a mechanically assisted instrument (MAI). Reported advantages of mechanically assisted versus manual manipulation include standardized thrust velocity and magnitude [1]. A systematic review reported clinically meaningful benefits for acute and chronic spinal pain, temporomandibular joint dysfunction, and trigger points in the trapezius muscles. However, the authors noted that the clinical trials reviewed suffered from multiple limitations, from study design to sample size and follow-up periods, as well as a lack of control or sham treatment groups, therefore limiting the external validity of their observations [2]. More recently, a prospective, randomized, blinded, placebo-controlled study showed benefits in terms of reduced frequency of painful episodes (neck pain) and improved internal rotation strength, but no significant differences in severity of pain at rest, proportion of patients reporting pain with active movement, neck stiffness, or shoulder impingement on internal rotation [2].

Reported complications are rare. A case report of a cerebral hemorrhage following chiropractic activator treatment was published in 2016 [3]. We report a case of neuropathic pain following treatment with the Activator device. While transient neuropathic pain may be considered by most spinal practitioners as a mild complication, the elderly are at risk of secondary complications as changes in steroids, and NSAID doses can be associated with complications ranging from insomnia, confusion, and adrenal gland dysfunction to nephrotoxicity, respectively. Further, a reduction in mobility is associated with complications in elderly patients [4]. Here, objective mobility data were collected as described by Ahmad et al. [5], demonstrating a significant reduction in the average number of daily steps post-intervention [6-8]. The patient suffered emotionally with frustration over his pain and disability as well as having to miss several prescheduled family activities.

## Conclusions

Complications of mechanically assisted manipulation are rarely reported, and the authors believe that this case will raise awareness of potential risks, particularly in the elderly. Manipulative treatment may not have been appropriately indicated in this case. Future studies aimed at clarifying indications for manipulative treatments are needed, as this report serves as an example that even relatively safe treatments may still create significant adverse effects.
